# COVID-19 Vaccine Perspective From Adolescents’ Lens in the US

**DOI:** 10.7759/cureus.53566

**Published:** 2024-02-04

**Authors:** Meher Garg, Arnav A Nagrecha, Ruchi Gupta, Makeba Williams

**Affiliations:** 1 Physician Preparatory Pathway Program, Springfield High School and Southern Illinois University (SIU) School of Medicine, Springfield, USA; 2 College of Science and Engineering, University of Minnesota, Minneapolis, USA; 3 Internal Medicine, Springfield Clinic, Springfield, USA; 4 Obstetrics and Gynaecology, Washington University School of Medicine, St. Louis, USA

**Keywords:** covid-19 vaccine hesitancy in adolescents, covid-19 vaccine acceptance in adolescents, covid 19 booster in adolescents, parental influence on covid 19 vaccination, covid 19 vaccine

## Abstract

Introduction

The COVID-19 pandemic has presented an unprecedented global health issue. The World Health Organization estimates 773 million confirmed cases and 7 million deaths. Vaccination continues to be the most effective way to prevent COVID-19 and has demonstrated safety and efficacy in all age groups. Though a lot of studies have looked at COVID-19 vaccination acceptance and hesitancy in adults, there is scarce research addressing adolescent vaccination readiness. COVID-19 infection in this age group may result in lost school days, school and community transmission, and loss of productivity for parents.

Aim

This study aims to determine COVID-19 vaccination rates and factors influencing its acceptance and hesitancy in adolescents in a community setting.

Methods

A voluntary survey was conducted at a local high school in May 2023. Information was collected about the demographics of adolescents and the educational background of parents/guardians. The survey assessed the COVID vaccine rate, reasons for COVID-19 vaccine acceptance or refusal, number of doses of COVID-19 vaccine and boosters received, prior history of COVID-19 infection, source of information on COVID-19 vaccine, flu vaccine acceptance by the students, and whether they would be willing to take a COVID-19 vaccine booster.

Results

Four hundred participants, ranging in age from 13 to 19, were surveyed. The vaccination rate in boys was comparable to that in girls. 72% received at least one COVID-19 vaccine, and 66% were considered completely vaccinated. Of those completely vaccinated, 80% had undergone further updated COVID-19 booster vaccinations. Adolescents whose parents/guardians were college graduates had a higher vaccination rate than those whose parents/guardians were not. Caucasians and Asians had a higher vaccination rate compared to African Americans and mixed races. The vaccination rate was not statistically different in adolescents with prior COVID-19 infection versus no prior infection. Flu vaccination was associated with higher COVID-19 vaccination rates. Lack of trust was an important reason for vaccine hesitancy, along with questions about efficacy, concerns about side effects, parent/guardian decisions, and religious reasons. Protecting oneself, family/friends, and community were the major reasons to take the vaccine. Parents/guardians, physicians, peers, television, social media, flyers, and schools were the primary sources that adolescents relied on for information about the COVID-19 vaccination.

Conclusion

Lower education attainment among parents/guardians, African Americans, and mixed races was associated with lower vaccination rates. Lack of trust in the vaccine, questions about efficacy, and fear of side effects were the most frequently cited reasons for vaccine hesitancy. Parent/guardian influence and religious reasons were other significant reasons for vaccine hesitancy. Flu vaccination was associated with higher COVID-19 vaccination rates. Understanding factors influencing COVID-19 vaccination will allow us to address barriers to COVID-19 vaccination and other vaccinations appropriate for this age group. Educating adolescents in schools, involving local and state health departments to increase awareness about the vaccine, and educating parents and guardians along with the teenagers can help increase the acceptance of the vaccine. These interventions will also positively affect the acceptance of the booster and prepare us for any future pandemics.

## Introduction

Coronavirus disease, also known as COVID-19, is a respiratory disease caused by the SARS-CoV-2 virus. It was first discovered in December 2019 in Wuhan, China. The World Health Organization (WHO) declared it a public health emergency and later a pandemic [1,2]. As per the WHO, there have been 773 million confirmed cases and 7 million deaths globally [2]. Over 6.7 million hospitalizations and 1.16 million COVID-19-related deaths have been reported in the United States by the Centers for Disease Control and Prevention (CDC) [2,3]. The Food and Drug Administration (FDA) approved the COVID-19 vaccine on December 11, 2020, for ages 16 and older [1,3,4]. The FDA authorized emergency use of the COVID-19 vaccine in children aged 12-15 years in May 2021 and in children aged 5 through 11 years in October 2021 [1,3,4]. Vaccination continues to be the most effective way to prevent COVID-19 and its severe consequences, and it has demonstrated safety. One major challenge to vaccination success is the phenomenon of vaccine hesitancy. Vaccine hesitancy can be defined as the reluctance or rejection of getting the vaccines despite the availability of vaccination services [5]. The potential factors that can drive vaccine hesitancy are mostly related to a lack of confidence in the safety and effectiveness of vaccination, as well as inconvenience and complacency.

Vaccination rates vary with age and are lowest in the pediatric and adolescent populations. As of May 11, 2023, approximately 94% of adults older than 65 living in the US have completed their primary vaccination series (CDC reference). However, the vaccination rate was 83.8% in the age group 50-64 years and 72.2% in the age group 25-29 years [3]. As per the American Academy of Pediatrics and CDC, 17.8 million US children and adolescents ages 12-17 have received at least one dose of the COVID-19 vaccine, representing 72.2% of 12- to 17-year-olds, and 16 million US adolescents ages 12-17 have completed the two-dose vaccination series, representing 61.8% of that age group [3].

Unequal global distribution and the continued emergence of SARS-CoV-2 variants are possible challenges to successful COVID-19 vaccination [5]. Factors influencing vaccine acceptance in adults have been studied and include ethnicity (African Americans had a lower acceptance); working status (unemployed people had a lower acceptance); gender (women had a lower acceptance); education (participants with low education had a lower acceptance); age (younger age was associated with a lower willingness to receive vaccination); work status (workers in healthcare settings are more likely to be immunized; concern (those who were highly concerned about being infected were less likely to refuse the vaccine); personal, religious, or political beliefs; and those who received vaccinations (especially influenza) in the past had a higher acceptance [5]. 

Though a lot has been studied about COVID-19 vaccination in adults, vaccination rates and factors that may influence vaccine acceptance and hesitancy in adolescents need further understanding. Although children and adolescents are generally at lower risk of severe infection, they do experience emotional distress, morbidity, and mortality. The purpose of this study is to determine COVID-19 vaccination rates in adolescents and the factors influencing COVID-19 vaccine acceptance and hesitancy in this age group in a community setting.

## Materials and methods

A questionnaire was developed to include demographic information about the adolescents, such as their age, gender, race, and educational background of parents/guardians. Information was collected on COVID-19 vaccine acceptance or refusal, reasons for vaccine acceptance/refusal, number of doses of COVID-19 vaccine taken, prior history of COVID-19 infection in students and family members, source of information on COVID-19 vaccine, flu vaccine acceptance by the students, and if they would be open to taking the COVID-19 booster vaccine yearly if that became a recommendation. No participant identifiers were requested in the questionnaire. The study was reviewed by the institutional research committee and was IRB-exempt.

The voluntary questionnaire was distributed to adolescents in the public high schools in Springfield, IL, from May 8 to May 22, 2023. The students were allowed to discuss the questionnaire with their parents or guardians (Table 1).

**Table 1:** Questionnaire

Questionnaire
Age
Race
Highest level of education of parents/guardians
Prior COVID infection in oneself or family/household
COVID-19 vaccine taken
If COVID-19 vaccine accepted how many doses, including booster
Reason(s) for taking the COVID-19 vaccine
Reason(s) for not taking the COVID-19 vaccine
Sickness/side effects from COVID-19 vaccine, if taken
Source of information for the COVID-19 vaccine
Flu vaccine acceptance
Open to taking COVID-19 vaccine yearly

The survey was closed after 400 responses were obtained. The survey findings were compiled without any identifying information. Statistical analysis was performed using SPSS version 23 (IBM Corp., Armonk, NY). A paired t-test was used to calculate the odds ratio, 95% confidence interval, and p-values.

## Results

Four hundred adolescents between the ages of 13 and 19 (mean 16.01) were surveyed. The gender breakdown was 220 boys and 180 girls. The demographic breakdown was Caucasian (61%, 244/400), Asian (10%, 40/400), African American (16%, 64/400), mixed race (9%, 36/400), and students who did not wish to state their race (4%, 16/400) (Table 2).

**Table 2:** Demographic data of the study population

Individual variables	Number	Percent
Age	13–19 years	Mean 16.01
Gender		
Male	220	55%
Female	180	45%
Ethnicity		
Caucasian	244	61%
African American	64	16%
Asian	40	10%
Hispanic	4	1%
Mixed race	36	9%

**Figure 1:**
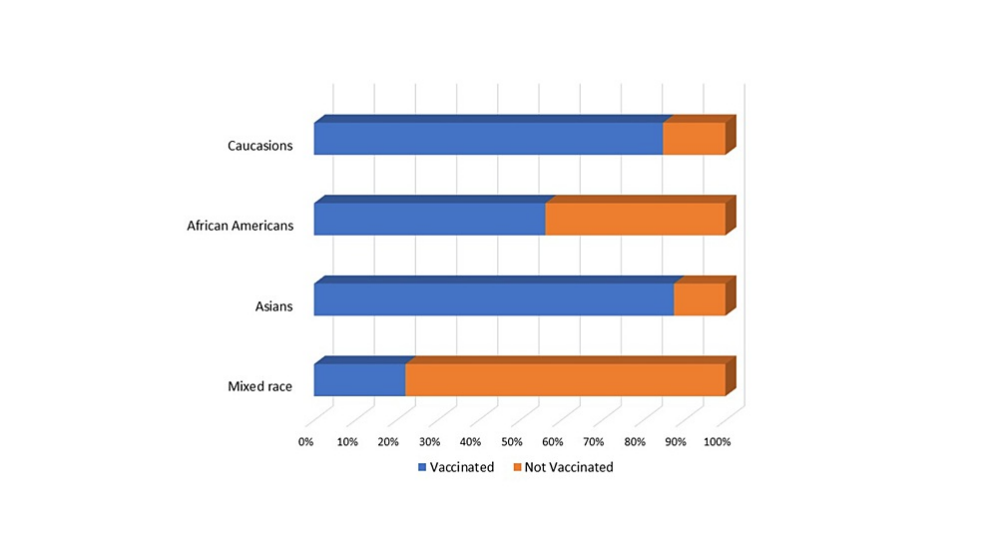
Vaccination data by ethnicity

**Figure 2:**
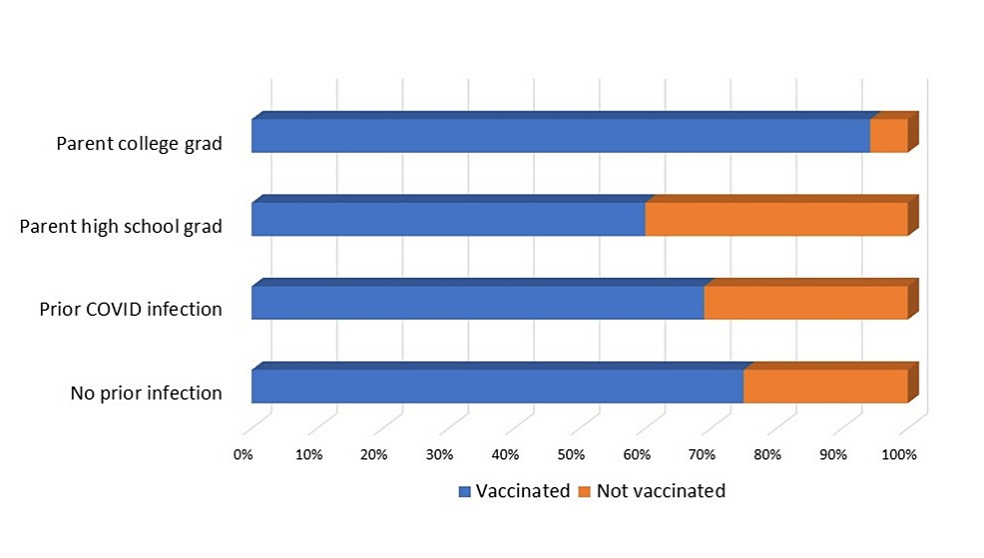
Vaccination data by parental/guardian education and by history of prior COVID-19 infection

The vaccination rate in boys was 73% (160/220), which was comparable to girls 71% (128/180); 72% (288/400) had at least 1 COVID-19 vaccine. 66% (264/400) were considered completely vaccinated (at least two doses); 53% (212/400) had taken at least one booster. Of the students who were considered completely vaccinated (at least two doses), 80% (212/264) had undergone further COVID booster vaccinations. Thirty-five percent (140/400) stated their parents or guardians are college graduates. Adolescents whose parents/guardians are college graduates had a vaccination rate of 94% (132/140) compared to 60% (156/260) whose parents are not college graduates, OR 1.57 (CI: 1.15-2.14, p=0.004). Caucasian (85%, 208/244) and Asian (90%, 36/40) had a higher vaccination rate compared to African Americans (56%, 36/64) and those who were mixed race (22%, 8/36), OR 1.91 (CI: 1.29-2.83, p=0.001). Fifty-eight percent (232/400) recalled that they had tested positive for COVID-19, and 84% (336/400) students stated that someone in their household had COVID-19. The vaccination rate was 69% (160/232) in teenagers with prior COVID-19 infection versus 76% (128/168) with no prior infection, OR 0.90 (CI: 0.67-1.22, p=0.5). Of the students surveyed, 57% (228/400) consider taking the COVID-19 vaccine booster yearly, and 62% (248/400) students stated that they take the yearly flu vaccine, OR 0.91 (CI: 0.73-1.15, p=0.47). However, adolescents who take the flu vaccine yearly had a higher vaccination rate for COVID-19 at 81% (200/248) compared with the vaccination rate of 58% (88/152) who do not take the yearly flu vaccine, OR 1.39 (CI: 1.01-1.92, p=0.04) (Figures 1-2 and Table 3).

**Table 3:** Vaccination data Vaccination data based on gender, race, parental education, prior history of COVID-19 infection, history of flu vaccination

Individual variables	Number	Vaccinated	Percent	Odd ratio, confidence interval
Gender				OR-1.02, CI: 0.75–1.38
Male	220	160	73%	
Female	180	128	71%	
Race/ethnicity				OR-1.91, CI: 1.29–2.82, p=0.001
Caucasian	244	207	85%	
African American	64	36	56%	
Asian	40	35	88%	
Mixed	36	8	22%	
Education background of parent/guardian				OR-1.57, CI: 1.15–2.14, p=0.004
College graduates	140	132	94%	
High school	260	156	60%	
Vaccination based on prior infection				OR-0.9, CI: 0.67–1.22, p=0.5
Prior COVID infection	232	160	69%	
No prior COVID infection	168	120	76%	
Vaccination based on Flu vaccination				OR-1.39, CI: 1.01–1.92, p=0.04
Take flu vaccine	248	200	81%	
Do not take the flu vaccine	152	88	58%	

For vaccine hesitancy, the major factors were: do not trust (57%, 64/112), question efficacy (21%, 24/112), worried about side effects (18%, 20/112), parent or guardian decision (39%, 44/112), religious (12%, 14/112), and others (11%, 12/112). The major reasons to take the vaccine (the participants were allowed to pick more than one response) were to protect themselves (74%, 213/288), protect family/friends (66%, 190/288), protect the community (13%, 37/288), and other reasons (11%, 32/288). The primary sources of information for the COVID-19 vaccine were parents or guardians (73%, 292/400) and physician offices (40%, 161/400). Other sources of information for the COVID vaccine were peers (19%, 77/400), television (14%, 57/400), social media (12%, 49/400), flyers (2%, 8/400), and schools (3%, 12/400). The majority had used multiple sources of information for the vaccine (Table 4).

**Table 4:** Factors for COVID-19 vaccine acceptance and hesitancy Reasons for COVID-19 vaccine acceptance and hesitancy and source of information for the vaccine

	Number of adolescents (can pick more than one response)	Percentage
Reasons for taking COVID-19 vaccine		
To protect self	224	78%
To protect family/friends	212	74%
To protect community	72	25%
Other reasons	20	7%
Reasons for COVID-19 vaccine hesitancy		
Do not trust	64	57%
Question efficacy	24	21%
Worried about side effects	20	18%
Parent/guardian decision	44	39%
Religious	14	12%
Others	12	11%
Source of information for COVID-19 vaccine		
Parent/guardian	120	30%
Peers	28	7%
Physician offices	16	4%
Social media	16	4%
All of the above	216	54%

Out of 288 students who had the COVID-19 vaccine, 48% reported no side effects from it, whereas 50% had mild/minimal symptoms. Only six (2%) students reported severe symptoms from the vaccine.

## Discussion

Although the COVID-19 disease course is milder in adolescents than in adults, it is important to include adolescents as targets for vaccination to prevent the spread to more vulnerable populations [6,7]. Vaccination readiness and vaccine hesitancy are key factors for vaccination coverage. Though a lot of studies have looked at COVID-19 vaccination acceptance and hesitancy in adults, there is scarce research addressing adolescent vaccination readiness.

Our study surveyed 400 adolescents aged 13-19 years and found that the vaccination rate was 72%, comparable to the data published by the Center for Disease Control in 12- to 17-year-olds. The adolescents completing the primary two-dose vaccination series were 66% (264/400) compared to CDC data of 61.8% in 12- to 17-year-olds. The comparative rate of having completed the primary two-dose immunization in adults, as reported by the CDC, in 25-49 years old is 72.2% and increased to 83.8% in the age group 50-64 years and is highest at 94.4% in individuals older than 65 years.

Brandt et al. assessed adolescents’ attitudes towards COVID-19 vaccination in the US prior to COVID-19 vaccine approval in adolescents [8]. They concluded that although 76% of the respondents in the age group 14-24 years reported a willingness to get vaccinated, many had concerns about side effects, efficacy, and safety and would wait until they believed it was safe and recommended by experts. A similar study in Canada of 16-21-year-olds conducted between November and December 2020 observed a vaccination willingness of 65.4%, with no difference by age, but sociodemographic characteristics such as household income had a negative impact on vaccine willingness [9]. One major finding of the study was that the education status of participants did affect their willingness to receive vaccination, with high school students reporting a higher willingness than school dropouts of the same age [9]. This is in line with results from a systematic review by Lin et al., showing lower vaccine acceptance among less-educated adults [10].

In our study, Caucasians (85%, 207/244) and Asians (88%, 35/40) had a higher vaccination rate compared to African Americans (56%, 36/64) and mixed race (22%, 8/36), OR - 1.91 (CI: 1.29- 2.83, p-0.001). The CDC vaccine data reports vaccination rates by ethnicity only for the adult population. As per the CDC data, Asians had the highest rate of completing the primary two-dose vaccination schedule at 97%. Native Americans had the lowest rate of 75.8%. Caucasian, African American, and Hispanics had similar rates of completing the primary vaccination rate of 83-85% [3].

There was no gender difference in the vaccination rate in our study. Bono et al. reported that gender did influence vaccine acceptance, with lower vaccination willingness in females, likely due to their perceived fear of side effects [11]. A systematic review and meta-analysis by Robinson et al. revealed that being female, having a young age, having a lower income or education level, and having a migrant background impacted negatively on the intention to vaccinate [12]. Schuiteman et al. reported that gender did influence vaccine acceptance, with male adolescents showing a higher willingness to vaccinate [13]. However, a similar study by Aff et al. found no difference in vaccine willingness by gender [9].

In our study, parents and guardians were the most important sources of information and had an influence on vaccine acceptance. Eighty-four percent of adolescents relied on parents/guardians as the source of information for the vaccine. Parents/guardians' decision (36%) was the second most important factor for not accepting the vaccination. Further, adolescents whose parents/guardians are college graduates had a vaccination rate of 94% (132/140) compared to 60% (156/260) whose parents are not college graduates, OR - 1.57 (CI: 1.15-2.14, p=0.004). A systematic review and meta-analysis by Chen et al. looked at the worldwide vaccination acceptance rate among parents and guardians for the COVID-19 vaccine for their children [14]. However, this meta-analysis did not stratify the children further into age groups. During the determinant assessment, researchers observed that the age of parents and guardians, access to scientific information and recommendations, and the willingness of parents and guardians to vaccinate themselves potentially predicted vaccination acceptance. The education level and gender of parents and guardians were not conclusive predictors. Contradictory results were reported on the association between high-level education and vaccination acceptance [14]. Higher education gave parents and guardians access to more and better information about the virus and vaccine, providing them with more tools for decision-making.

Adolescents in our study who were up-to-date on their immunization schedule or had a history of taking the seasonal influenza vaccine were more likely to be vaccinated against COVID-19. Adolescents who take the yearly flu vaccine had a higher vaccination rate for COVID-19 at 81% (200/248), compared with a vaccination rate of 58% (88/152) who do not take the yearly flu vaccine, OR - 1.39 (CI: 1.01-1.92, p=0.04). Further, of the students surveyed, 57% considered taking a COVID-19 vaccine booster yearly, if required, and 62% of students stated that they take the flu vaccine yearly. Galarce et al. observed during the H1N1 pandemic that those who had previously received the influenza vaccine were 21 times more likely to receive the H1N1 vaccine [15]. In the systematic review and meta-analysis by Chen et al., routine influenza vaccination behavior was observed to be a potentially significant predictor of vaccination acceptance for COVID-19 [14].

In our study, 48% reported no side effects from the vaccine, and 144 (50%) reported only mild symptoms. The most commonly reported reactions are dizziness, vaccination/injection site pain, pyrexia, headaches, myalgias, and fatigue [16]. Occasional cases of myocarditis and pericarditis in children and adolescents have been reported after the administration of the mRNA COVID-19 vaccine; though the published literature explores a possible association between the COVID-19 vaccine and myocarditis, the incidence is too small to provide a causal association [17].

The CDC currently recommends one dose of an updated COVID-19 booster for ages six months and older. The CDC reports a booster vaccination rate of 7.8% in the age group 12-17 years, 12.3% in 25-49-year-olds, and it increases to 21.7% in the age group 50-64 years and is highest at 43.3% in 65 years and older [3]. In our study, 40% (160/400) of the adolescents had at least 1 updated booster, compared to CDC data of 7.8%. Adolescents who completed the primary vaccination series had a 61% rate (160/264) of taking the booster dose.

While interpreting the results, the following limitations should be considered: first, the sample might not be representative of all adolescents. Second, the student sample was rather small. Third, vaccination willingness and perception could change with time, and this was not assessed as follow-up.

## Conclusions

Lower education levels of parents/guardians, African Americans, and people of mixed races were associated with vaccine hesitancy. Lack of trust, questions regarding the vaccine's efficacy, and parental influence were the main reasons for declining the vaccine. Prior flu vaccination increased the likelihood of adolescents accepting the COVID-19 vaccine.

By understanding factors influencing COVID-19 vaccination hesitancy, we can understand and work on barriers to the COVID-19 vaccine, booster, and other vaccinations offered in this age group. Educating adolescents in schools, involving local and state health departments to increase awareness, and educating parents and guardians can help increase the acceptance of the vaccine. More efforts may be required to improve adolescents' confidence and willingness to vaccinate compared to adults. These interventions will also positively affect the vaccine willingness for the updated booster and prepare us for any future pandemics.
